# Exploring the association of adverse drug reactions with medication adherence and quality of life among hypertensive patients: a cross-sectional study

**DOI:** 10.1007/s11096-024-01832-9

**Published:** 2024-11-28

**Authors:** Widya N. Insani, Li Wei, Rizky Abdulah, Sofa D. Alfian, Nurul A. Ramadhani, Rizky Andhika, Neily Zakiyah, Matthew Adesuyan, Yunisa Pamela, Rima Mustafa, Cate Whittlesea

**Affiliations:** 1 Department of Pharmacology and Clinical Pharmacy, Padjadjaran University, Bandung, Indonesia; 2 Centre of Excellence for Pharmaceutical Care Innovation, Padjadjaran University, Bandung, Indonesia; 3 Research Department of Practice and Policy, School of Pharmacy, University College London, London, UK; 4 Centre for Medicines Optimisation Research and Education, University College London Hospitals NHS Foundation Trust, London, UK; 5 Division of Nephrology and Hypertension, Department of Internal Medicine, Faculty of Medicine, Padjadjaran University, Hasan Sadikin General Hospital, Bandung, Indonesia; 6 Department of Biomedical Sciences, Faculty of Medicine, Padjadjaran University, Bandung, Indonesia; 7 Cambridge Institute for Medical Research, University of Cambridge, Cambridge, UK; 8 Department of Epidemiology and Biostatistics, Imperial College London, London, UK

**Keywords:** Adverse drug reactions, Hypertension, Medication adherence, Quality of life

## Abstract

**Background:**

Effective hypertension management requires medication adherence to prevent complications. However, adverse drug reactions (ADRs) can undermine adherence and negatively affect patients’ quality of life. Limited research has explored the association between ADRs, medication adherence, and health-related quality of life (HRQoL) in individuals with hypertension.

**Aim:**

To investigate the association between ADRs, medication adherence, and HRQoL among patients with hypertension.

**Method:**

A cross-sectional study using telephone interviews and medical record reviews was conducted in 11 primary care facilities in Indonesia. The causality of reported ADRs was assessed using the Naranjo algorithm, validated by a panel of experts in pharmacy practice and medication safety. The severity of ADRs was classified using the Hartwig scale. Adherence to antihypertensive drugs was estimated using the Medication Adherence Report Scale-5 (MARS-5). The EuroQoL EQ-5D-5L was used to measure HRQoL. The association between ADRs and medication adherence was assessed using multivariate logistic regression, while the association with HRQoL was evaluated through the Tobit regression model.

**Results:**

A total of 507 patients were included in this study. We found that 20.32% (n = 103) of the patients experienced ADRs, with the most commonly reported ADRs being polyuria and urgency, gastrointestinal symptoms, leg swelling, dizziness/hypotension, palpitations, and dry cough. The majority experienced mild ADRs (n = 75, 72.82%), while 27.18% (n = 28) had reactions of moderate severity. Experiencing ADRs was associated with reduced medication adherence (adjusted odds ratio (OR) 7.15, 95% CI 4.07–12.55) and decreased HRQoL (coefficient: − 0.037).

**Conclusion:**

Patients experiencing ADRs were seven times more likely to be non-adherent to their medication regimen and reported a reduced quality of life compared to those without ADRs, placing them at a higher risk of suboptimal treatment outcomes. This finding highlights the need for additional monitoring and education for patients affected by ADRs, particularly through more frequent clinical and laboratory assessments, timely management of ADRs, and personalized education on the importance of adherence to prevent hypertension-related complications.

**Supplementary Information:**

The online version contains supplementary material available at 10.1007/s11096-024-01832-9.

## Impact statements

- Patients with ADRs are seven times more likely to be non-adherent to their antihypertensive regimen and report a lower quality of life compared to those without ADRs.
- Patients affected by ADRs should be monitored more closely, as they are at a higher risk of suboptimal treatment outcomes.
- Additional monitoring and education should include more routine clinical and laboratory assessments, timely management of ADRs, and an emphasis on the importance of medication adherence.

## Introduction

Hypertension is the leading modifiable risk factor for cardiovascular disease (CVD)-related morbidity and mortality, affecting 1 billion people worldwide [1]. Approximately 31.1% of global adult population has hypertension, with the prevalence increasing in low- and middle-income countries (LMICs) [2]. Previous studies have shown that 55.4% to 61.7% of patients with hypertension worldwide had uncontrolled blood pressure, which indicates a significantly increased risk of CVD and related complications [3, 4].

Medication adherence is crucial in managing chronic diseases such as hypertension for effective disease control [5]. A previous systematic review revealed that up to 40% of patients were non-adherent to their antihypertensive regimen worldwide [6]. Poor medication adherence has been linked to CVD, including myocardial infarction and stroke; impaired renal function, and all-cause mortality [7]. Several factors may hinder medication adherence, including treatment-related factors, such as the occurrence of adverse drug reaction (ADRs) [8].

The prevalence of ADRs to antihypertensive drugs ranges from 16 to 40%, with symptoms including dizziness, electrolyte imbalances, gastrointestinal symptoms, leg swelling, flushing, headaches, dry cough, urinary incontinence, and sexual dysfunction [9–13]. Tsang et al. found that antihypertensive drugs were the most commonly implicated drug class in ADRs in the primary care setting [14]. A previous review also showed that ADRs to antihypertensive drugs were among the most common causes of ADR-related hospital admissions, highlighting the significant burden of these iatrogenic complications for patients [15, 16].

Current research on ADRs related to antihypertensive drugs has predominantly focused on their frequency, with relatively few studies exploring their impact on patients, particularly in relation to medication adherence and health-related quality of life (HRQoL) [12, 17]. This is particularly critical in LMICs, including Indonesia, where 80% of the global burden of CVD-related deaths occurs, in contrast to developed countries [10, 11]. Such information could improve our understanding of the overall burden of ADRs on patients and the healthcare system, and inform the implementation of strategies to improve medication safety and adherence for patients with hypertension.

### Aim

This study aimed to investigate the association between ADRs from antihypertensive drugs and (i) medication adherence and (ii) HRQoL among patients with hypertension.

### Ethics approval

This study was approved by the Research Ethics Committee of Universitas Padjadjaran, Indonesia (No.18/UN6.KEP/EC/2022;11/02/2022) and the Research Ethics Committee of University College London, United Kingdom (project ID: 22,711/001;02/08/2022).

## Method

### Study design and population

This was a cross-sectional study using telephone interviews and medical record reviews among patients with hypertension in 11 primary care facilities in Indonesia. The study population consisted of patients with hypertension who visited the clinics for consultation during the study period. The inclusion criteria were patients aged ≥ 18 years with a diagnosis of hypertension and who were prescribed an antihypertensive drug. We excluded patients who were pregnant, in the lactation period, or unable to communicate in the Indonesian language. Considering an estimated prevalence of non-adherence to antihypertensive drugs of 45.5% [18], with a 95% CI and a margin of error of 0.05, the required minimum sample size was 382 patients.

Eligible patients were screened with the help of primary care staff. During visits, prospective participants received study information and were invited to participate in the study. Those who consented were asked to sign an informed consent form and provide their telephone number. Subsequently, the lead researchers contacted the patients to schedule an interview time and conducted the telephone interview on ADRs, medication adherence, and HRQoL. In a few cases, such as those with hearing difficulties, the interviews were completed through text messages.

### ADR

An ADR is defined as “a response to a drug that is noxious and unintended, and which occurs at doses normally used in humans for prophylaxis, diagnosis, or therapy of disease, or for the modification of physiological function” [19], such as leg swelling related to calcium channel blockers (CCBs) and dry cough related to angiotensin-converting enzyme inhibitors/angiotensin receptor blockers (ACEIs/ARBs). A questionnaire developed by Chrischilles et al. was used in this study to obtain information on ADRs [20]. Patients were asked if they had experienced any side effects, unwanted reactions, or other issues from their antihypertensive medication in the past six months. A six-month period was chosen to minimize potential recall bias, as longer recall periods may lead to inaccurate reporting [21]. Those who responded affirmatively were then asked if they had discussed the symptom with their doctor. They were further asked whether: (i) The doctor told them to continue the medication as before; (ii) The doctor changed the medication or prescription; (iii) The doctor prescribed another drug to address the symptom or provided advice; (iv) The doctor ordered lab tests; (v) Other actions. Patients who did not discuss the symptom with their doctor were asked if they: (i) Continued the medication; (ii) Changed the dose/schedule on their own; (iii) Temporarily or (iv) Permanently stopped the medication; (v) Took other actions. Patients were asked if the symptom persisted after continuing or discontinuing the medication, based on their previous responses [20, 22, 23]. Patients were also asked if the symptom had led to an emergency room or hospital admission, if they had experienced similar symptoms with previous antihypertensives, and how much the symptom impacted their daily activities [20].

Subsequently, the causality of the reported ADRs was assessed by a research pharmacist (WNI) using the Naranjo ADR Causality Algorithm [22]. To ensure robust categorization, 10% of randomly selected cases were validated by a panel of experts in pharmacy practice and medication safety. The panel then met to discuss ADRs and exclude doubtful cases. The severity of ADRs was classified using the Hartwig Severity Scale. Mild ADRs were defined as reactions not requiring a change in medication, moderate ADRs involved changes in treatment or hospitalization, and severe ADRs required intensive medical care, caused permanent harm, or were linked to patient death [24].

The forward–backward translation process, as recommended by the World Health Organization (WHO), was used to translate the questionnaire from English to Indonesian (*Bahasa*). First, two Indonesian research pharmacists (WNI and SDA), fluent in English and knowledgeable in ADR terminology, independently performed the forward translation. Two Indonesian physicians (YP and RM) then reviewed the draft for practicality and context. The bilingual pharmacist (NZ), unaware of the original questionnaire, translated the Indonesian version back into English to ensure unbiased translation. Finally, a native English-speaking pharmacist (MA) compared the translation with the original English questionnaire to confirm conceptual equivalence. Following forward–backward translation process, a pilot survey was conducted among 10 hypertensive patients and healthcare professionals to ensure comprehensibility. The results indicated no changes to the content of the items.

### Medication adherence

Medication adherence was assessed using the Medication Adherence Report Scale (MARS-5) [25]. The MARS-5 has been translated into and validated in the Indonesian language, with a Cronbach’s α coefficient of 0.803, indicating good internal consistency [18, 26]. The MARS-5 consists of five statements: four reflecting intentional non-adherence (stopping, changing the dose, taking less than prescribed, skipping doses) and one addressing unintentional non-adherence (forgetting to take the medication). The MARS-5 score ranges from 5 to 25, with non-adherence defined as a score of less than 80% of the total [27].

Three subgroup analyses were performed to compare determinants of intentional, in-part intentional, and unintentional non-adherence, as classified by Alfian et al. [18] Firstly, *intentional non-adherence* refers to patients who report some form of intentional non-adherence (score 1–3 for at least one of items 2–5) while remaining adherent on the unintentional non-adherence item (score 4–5 for item 1). Secondly, *partial intentional non-adherence* refers to patients who report non-adherence on both intentional (score 1–3 for at least one of items 2–5) and unintentional non-adherence items (score 1–3 for item 1). Third, *unintentional non-adherence* refers to patients who report being non-adherent on unintentional adherence (score 1–3 for item 1) but adherent on all intentional non-adherence items (score 4–5 for items 2–5). The subgroup classification could result in patients previously considered adherent being reclassified into a non-adherence subgroup based on their responses, highlighting the complexity of adherence behavior [18, 28].

### HRQoL

HRQoL was assessed using the EuroQoL EQ-5D-5L [29]. The EuroQoL EQ-5D-5L consists of five questions, each representing one of the five HRQoL dimensions: mobility, self-care, usual activities, pain/discomfort, and anxiety/depression. Each dimension is rated on a scale from 1 to 5 [29]. HRQoL measurement depends on the societal values of the target population, which may reflect the specific standard of living preferences of that population [30, 31]. In this study, the Indonesian EQ-5D-5L value set, which has been previously validated, was used to calculate the HRQoL score [32].

### Covariates

The covariates included age, gender, duration of hypertension, class of antihypertensive medication, the presence of polypharmacy (defined as the use of ≥ 5 medications) [33], history of CVD, including myocardial infarction, stroke, transient ischaemic attack (TIA), and angina, presence of comorbidities, i.e., diabetes mellitus, dyslipidemia, and hyperuricemia; smoking status, and education level.

### Data analysis

First, descriptive statistics were used to summarize patients’ characteristics, followed by chi-square tests to identify univariate associations with medication adherence. A multivariate logistic regression model was used to examine the association between ADRs and medication adherence. Sensitivity analysis was performed by including only ADRs categorised as probable based on the Naranjo causality algorithm. To address the ceiling effect of the EQ-5D utility score (44.77% in this study), the multivariate Tobit regression model was employed [34]. Subgroup analyses were conducted to identify determinants of problem in each health dimension using logistic regression model. In all models, ADRs were the primary independent variable, and patients’ characteristics were included as covariates. A *p*-value of ≤ 0.05 was considered statistically significant. All statistical analysis was performed using STATA 17 [35].

## Results

### General characteristics of participants

A total of 507 patients were included in this study (response rate: 83.70%). Most participants were female (n = 378, 74.56%), with a mean (± SD) age of 60.15 years (± 10.76). Most participants had been diagnosed with hypertension for more than a year (83.53%). Monotherapy with a CCB was the most commonly prescribed antihypertensive drug class (n = 350, 69.03%). Approximately 21.10% of patients were prescribed a combination of two drug classes (n = 107, such as a CCBs and ACEIs/ARBs (n = 69, 13.61%) or other combinations (n = 38, 7.50%). Around 8.48% of participants (n = 43) had a history of CVD. Approximately 19.72% of patients had type 2 diabetes mellitus, 19.53% had dyslipidemia, and 10.45% had hyperuricaemia (n = 53). In the univariate analysis, older age was associated with higher medication adherence (*p*<0.001). Different antihypertensive drug classes also influenced adherence, with patients on monotherapy, particularly calcium channel blockers (CCBs), showing higher adherence compared to those on drug combinations (*p* = 0.001). Additionally, patients with diabetes mellitus were more likely to adhere to their medication regimen compared to those without diabetes (*p* = 0.003). Conversely, the occurrence of ADRs significantly decreased adherence (*p*<0.001) (Table 1).

**Table 1:** General characteristics of participants

Characteristics	Total (n = 507, 100)	Adherent (%) (n = 389, 76.73)	Non-adherent (%) (n = 118, 23.27)	*P* value
Age				<0.001*
≤ 40 years	21 (4.14)	15 (3.86)	6 (5.08)	
41–50 years	78 (15.38)	46 (11.83)	32 (27.12)	
51–60 years	138 (27.22)	95 (24.42)	43 (36.44)	
61–70 years	182 (35.90)	153 (39.33)	29 (24.58)	
≥ 71 years	88 (17.36)	80 (20.57)	8 (6.78)	
Sex, Male (%)	129 (25.44)	101 (25.96)	28 (23.73)	0.625
Duration of hypertension				0.217
< 1 years	83 (16.37)	59 (15.17)	24 (20.34)	
1–5 years	183 (36.09)	139 (35.73)	44 (37.29)	
6–10 years	119 (23.47)	99 (25.45)	20 (16.95)	
> 10 years	122 (24.06)	92 (23.65)	30 (25.42)	
Antihypertensive drug class				0.001*
Monotherapy				
CCB	351 (69.23)	251 (64.52)	100 (84.75)	
ACEI/ARB	27 (5.33)	25 (6.43)	2 (1.69)	
Combination of 2 drug classes				
CCB and ACEI/ARB	69 (13.61)	61 (15.68)	8 (6.78)	
Other 2 drugs combinations	38 (7.50)	32 (8.23)	6 (5.08)	
Combination of ≥ 3 drug classes				
Polypharmacy	85 (16.77)	65 (16.71)	20 (16.95)	0.951
Diabetes mellitus	100 (19.72)	88 (22.62)	12 (10.17)	0.003*
Dyslipidemia	99 (19.53)	76 (19.54)	23 (19.49)	0.991
Hyperuricemia	53 (10.45)	38 (9.77)	15 (12.71)	0.360
CVD history	43 (8.48)	39 (10.03)	4 (3.39)	0.023*
Education level				0.078
Primary or no education	120 (23.67)	94 (24.16)	26 (22.03)	
Secondary education	305 (60.16)	225 (57.84)	80 (67.80)	
College/University	82 (16.17)	70 (17.99)	12 (10.17)	
Smoking statussss				0.376
Current smoker	59 (11.64)	41 (10.54)	18 (15.25)	
Former smoker	80 (15.78)	62 (15.94)	18 (15.25)	
Never smoker	368 (72.58)	286 (73.52)	82 (69.49)	
ADRs to antihypertensive medication	103 (20.32)	49 (12.60)	54 (45.76)	p<0.001*

*CCB* Calcium channel blocker, *ACEI* Angiotensin-converting enzyme inhibitor, *ARB* Angiotensin receptor blocke, *CVD* Cardiovascular disease, *MI* Myocardial Infarction, *TIA* Transient Ischaemic Attack, *ADRs* Adverse Drug Reactions

### ADRs to antihypertensive drugs

Around 20.32% of participants (n = 103) experienced an ADR to antihypertensive drug, with 2.96% (n = 15) experiencing more than one reaction. The most commonly reported ADRs were polyuria and urgency, nausea, vomiting and abdominal pain, dizziness/hypotension, leg swelling, palpitations, and dry cough (Table 2). The majority of these patients reported possible ADRs (n = 84, 81.55%), while the remaining had probable ADRs (n = 19, 18.45%). Approximately 40.78% of the patients with ADRs (n = 42) discussed their symptoms with their primary care providers or received educational information about ADRs. Around 72.82% of patients experienced reactions with mild severity according to the Hartwig scale (n = 75), while 27.18% had ADRs of moderate severity (n = 28). The most common ADRs associated with CCBs or their combinations were polyuria and urgency, leg swelling, and nausea, while ACEIs were primarily associated with dry cough.

**Table 2:** Types of adverse drug reactions to antihypertensive medication

Types of adverse drug reactions	Total n = 121 (%)	Naranjo causality category		
		Possible ADR n = 102 (%)		Probable ADR n = 19 (%)
Polyuria and urgency	68 (56.20)	53 (51.96)	15 (78.95)	
Nausea, vomiting, and abdominal pain	13 (10.74)	13 (12.75)	0 (0.00)	
Dizziness, hypotension	9 (7.44)	9 (8.82)	0 (0.00)	
Leg swelling	6 (4.96)	5 (4.90)	1 (5.26)	
Palpitation	5 (4.13)	5 (4.90)	0 (0.00)	
Dry cough	4 (3.31)	1 (0.98)	3 (15.79)	
Fatigue, drowsiness	4 (3.31)	4 (3.92)	0 (0.00)	
Insomnia	3 (2.48)	3 (2.94)	0 (0.00)	
Others*	5 (4.13)	5 (4.90)	0 (0.00)	

*Other ADRs: skin itch 1, cold sweat 1, vertigo 1, diarrhoea 1, bitter tongue 1

### ADRs and medication adherence

Multivariate logistic regression showed that patients who experienced ADRs were seven times more likely to be non-adherent to their antihypertensive medication compared to those who did not report an ADR (adjusted OR 7.15, 95% CI 4.07, 12.55). Other factors associated with medication adherence included age and education level. Compared to older individuals, patients aged 41–50 and 51–60 years were less likely to have good adherence. A higher education level (university education) was associated with better adherence (adjusted OR 0.40, 95% CI 0.16, 0.99) compared to primary education (Table 3). Sensitivity analysis revealed that when only patients with probable ADRs were included in the model, the adjusted OR decreased but remained significant (adjusted OR 3.05, 95% CI 1.04, 8.94). Three subgroup analyses were conducted to identify determinants of different types of non-adherence. ADRs related to antihypertensive drugs were associated with intentional and partially intentional non-adherence (adjusted OR 2.80, 95% CI 1.51, 5.17, and 4.05, 95% CI 2.33, 7.05, respectively), but not with unintentional non-adherence (adjusted OR 0.67, 95% CI 0.28, 1.56) (Table 4).

**Table 3:** Determinants of medication non-adherence

Characteristics	Crude odds ratio (95% CI)	Adjusted odds ratio (95% CI)
Age		
≤ 40 years	4.00 (1.21, 13.19)	2.38 (0.60, 9.43)
41–50 years	6.96 (2.96, 16.36)	4.17 (1.59, 10.91)*
51–60 years	4.53 (2.01, 10.19)	3.70 (1.50, 9.08)*
61–70 years	1.97 (0.86, 4.51)	1.45 (0.59, 3.52)
≥ 71 years	1.00	1.00
Sex, Male (%)	0.93 (0.58, 1.50)	1.28 (0.63, 2.59)
Duration of hypertension		
< 1 years	1.00	1.00
1–5 years	0.80 (0.45, 1.43)	1.02 (0.51, 2.03)
6–10 years	0.50 (0.25, 0.98)	0.47 (0.21, 1.05)
> 10 years	0.80 (0.43, 1.50)	1.45 (0.70 3.02)
Antihypertensive drug class		
*Monotherapy*		
CCB	4.04 (0.93, 17.60)	3.82 (0.69, 21.19)
ACEI/ARB	0.80 (0.10, 6.19)	0.98 (0.10, 9.62)
*Combination of 2 drug classes*		
CCB and ACEI/ARB	1.31 (0.26, 6.96)	0.88 (0.14, 5.66)
Other 2 drugs combination	1.88 (0.34, 10.21)	2.03 (0.30, 13.81)
*Combination of* ≥ *3 drug classes*	1.00	1.00
Polypharmacy	1.00 (0.58, 1.74)	1.50 (0.75, 2.98)
Diabetes mellitus	0.38 (0.20, 0.73)	0.50 (0.24, 1.04)
Dyslipidemia	1.05 (0.63, 1.76)	1.05 (0.57, 1.94)
Hyperuricemia	1.33 (0.71, 2.51)	1.46 (0.57, 3.13)
CVD history	0.31 (0.11, 0.89)	0.52 (0.15, 1.83)
Education level		
Primary or no education	1.00	1.00
Secondary education	1.31 (0.79, 2.16)	1.04 (0.57, 1.90)
College/University	0.62 (0.29, 1.31)	0.40 (0.16, 0.99)*
Smoking status		
Current smoker	1.00	1.00
Former smoker	0.66 (0.31, 1.42)	1.15 (0.45, 2.90)
Never smoker	0.65 (0.36, 1.22)	0.91 (0.41, 2.00)
ADRs to antihypertensive medication	5.75 (3.60, 9.19)	7.15 (4.07, 12.55)*

*CCB* Calcium channel blocker, *ACEI* Angiotensin-converting enzyme inhibitor, *ARB* Angiotensin receptor blocker, *CVD* Cardiovascular disease. *MI* Myocardial Infarction, *TIA* Transient Ischaemic Attack, *ADRs* Adverse Drug Reactions

**Table 4:** Subgroup analyses comparing determinants of intentional, in-part intentional, and unintentional non-adherence to antihypertensive medication

Characteristics	Non-adherence		
	Intentional	In-part intentional	Unintentional
	Adjusted OR (95% CI)	Adjusted OR (95% CI)	Adjusted OR (95% CI)
Age			
≤ 40 years	1.10 (0.18, 6.75)	3.08 (0.89, 11.89)	4.72 (0.98, 22.85)
41–50 years	2.14 (0.70, 6.47)	3.41 (1.31, 8.90)*	2.20 (0.63, 7.68)
51–60 years	1.70 (0.61, 4.71)	3.24 (1.32, 7.99)*	1.86 (0.61, 5.67)
61–70 years	2.15 (0.83, 5.59)	1.20 (0.49, 2.96)	1.38 (0.46, 4.14)
≥ 71 years	1.00	1.00	1.00
Sex, Male (%)	1.13 (0.52, 2.48)	0.95 (0.45, 1.99)	0.96 (0.39, 2.35)
Duration of hypertension			
< 1 years	1.00	1.00	1.00
1–5 years	1.18 (0.49, 2.80)	1.00 (0.49, 2.05)	1.05 (0.37, 2.96)
6–10 years	1.26 (0.50, 3.17)	0.54 (0.24, 1.23)	1.72 (0.61, 4.90)
> 10 years	1.54 (0.62, 3.87)	1.32 (0.62, 2.82)	1.39 (0.47, 4.08)
Antihypertensive drug class			
*Monotherapy*			
CCB	2.88 (0.52, 15.85)	1.91 (0.42, 8.76)	0.31 (0.06, 1.58)
ACEI/ARB	2.33 (0.30, 18.22)	0.29 (0.02, 3.64)	0.25 (0.28, 2.23)
*Combination of 2 drug classes*			
CCB and ACEI/ARB	0.86 (0.12, 6.03)	0.73 (0.14, 3.88)	0.43 (0.08, 2.38)
Other 2 drugs combination	1.87 (0.27, 12.81)	0.73 (0.12, 4.40)	0.09 (0.01, 1.12)
*Combination of* ≥ *3 drug classes*	1.00	1.00	1.00
Polypharmacy	1.39 (0.67, 2.89)	1.45 (0.71, 2.93)	0.98 (0.38, 2.51)
Diabetes mellitus	1.26 (0.63, 2.52)	0.20 (0.08, 0.55)*	1.07 (0.47, 2.43)
Dyslipidemia	1.49 (0.79, 2.79)	1.26 (0.68, 2.35)	1.07 (0.47, 2.43)
Hyperuricemia	1.97 (0.93, 4.21)	1.03 (0.47, 2.27)	0.21 (0.03, 1.55)
CVD history	1.95 (0.69, 5.50)	0.83 (0.25, 2.77)	0.35 (0.59, 2.10)
Education level			
Primary or no education	1.00	1.00	1.00
Secondary education	0.98 (0.50, 1.93)	0.90 (0.48, 1.67)	0.83 (0.35, 1.94)
College/University	0.69 (0.26, 1.82)	0.41 (0.16, 1.05)	1.87 (0.69, 5.06)
Smoking status			
Current smoker	1.00	1.00	1.00
Former smoker	0.49 (0.18, 1.30)	2.06 (0.78, 5.45)	0.61 (0.18, 2.03)
Never smoker	0.48 (0.21, 1.09)	1.20 (0.52, 2.78)	0.61 (0.23, 1.62)
ADRs to antihypertensive medication	2.80 (1.51, 5.17)*	4.05 (2.33, 7.05)*	0.67 (0.28, 1.56)

*CCB* Calcium channel blocker, *ACEI* Angiotensin-converting enzyme inhibitor, *ARB* Angiotensin receptor blocker, *CVD* Cardiovascular disease, *MI* Myocardial Infarction, *TIA* Transient Ischaemic Attack, *ADRs* Adverse Drug Reaction

### ADRs and HRQoL

Table Supplementary 1 summarises the distribution of health dimension problems and the EQ-5D utility scores. We found that pain/discomfort was the most frequently reported health dimension issue (n = 226, 44.58%), while self-care problems were the least reported (n = 17, 3.35%). The mean EQ-5D utility score for all patients in this study was 0.89 ± 0.17, ranging from − 0.29 to 1.00. Approximately 44.77% (n = 227) of patients did not report any problems in any health dimension, resulting in a utility score of 1.00. Multivariate Tobit regression showed that patients who experienced ADRs had a 0.037-point reduction in EQ-5D utility scores compared to those without ADRs (Table 5). Sensitivity analysis, which included only patients with probable ADRs, showed a larger decrease in HRQoL (coefficient: − 0.15). We conducted a subgroup analysis to investigate the determinants of each health dimension problem among patients with hypertension. Patients with hyperuricemia were more likely to report mobility-related problems (adjusted OR 2.25, 95% CI 1.13, 4.48), while those with a longer duration of hypertension were less likely to report anxiety-related problems compared to newly diagnosed patients (adjusted OR 0.39, 95% CI 0.16, 0.95), highlighting the need for early education and support to address anxiety and potential medication adherence issues in newly diagnosed individuals (Supplementary Table 2).

**Table 5:** Association between adverse drug reactions and EQ-5D utility value

Characteristics	Total (n = 507)	Tobit univariate analysis			Tobit multivariate analysis		
		Coefficient	SE	*P*-value	Coefficient	SE	*P*-value
Age							
≤ 40 years	21 (4.14)	− 0.010	0.04	0.809	− 0.008	0.04	0.854
41–50 years	78 (15.38)	− 0.044	0.03	0.087	− 0.033	0.03	0.217
51–60 years	138 (27.22)	− 0.014	0.02	0.544	− 0.011	0.02	0.621
61–70 years	182 (35.90)	− 0.029	0.02	0.170	− 0.028	0.02	0.195
≥ 71 years	88 (17.36)	Ref	Ref	Ref	Ref	Ref	Ref
Sex, Male (%)	129 (25.44)	0.031	0.017	0.063	0.011	0.02	0.617
Duration of hypertension							
< 1 years	83 (16.37)	Ref	Ref	Ref	Ref	Ref	Ref
1–5 years	183 (36.09)	0.008	0.02	0.717	0.010	0.02	0.647
6–10 years	119 (23.47)	0.014	0.02	0.540	0.017	0.02	0.470
> 10 years	122 (24.06)	0.008	0.02	0.749	0.002	0.02	0.919
Antihypertensive drug class							
*Monotherapy*							
CCB	351 (69.23)	0.009	0.04	0.811	0.020	0.04	0.611
ACEI/ARB	27 (5.33)	0.037	0.05	0.437	0.038	0.05	0.442
*Combination of 2 drug classes*							
CCB and ACEI/ARB	69 (13.61)	0.008	0.04	0.843	0.025	0.04	0.570
Other 2 drugs combination	38 (7.50)	0.053	0.04	0.226	0.060	0.05	0.187
*Combination of* ≥ *3 drug classes*	22 (4.34)	Ref	Ref	Ref	Ref	Ref	Ref
Polypharmacy	85 (16.77)	− 0.013	0.499	0.49	− 0.006	0.02	0.766
Diabetes mellitus	100 (19.72)	0.001	0.02	0.936	− 0.004	0.02	0.830
Dyslipidemia	99 (19.53)	− 0.031	0.02	0.093	− 0.030	0.02	0.106
Hyperuricemia	53 (10.45)	− 0.024	0.01	0.327	− 0.017	0.02	0.493
CVD history	43 (8.48)	0.019	0.79	0.479	0.012	0.03	0.689
Education level							
Primary or no education	120 (23.67)	Ref	Ref	Ref	Ref	Ref	Ref
Secondary education	305 (60.16)	0.023	0.02	0.490	0.009	0.03	0.621
College/University	82 (16.17)	0.020	0.02	0.387	0.013	0.03	0.586
Smoking status							
Current smoker	59 (11.64)	Ref	Ref	Ref	Ref	Ref	Ref
Former smoker	80 (15.78)	0.003	0.03	0.922	− 0.009	0.03	0.754
Never smoker	368 (72.58)	− 0.031	0.02	0.179	− 0.030	0.03	0.236
ADRs to antihypertensive medication	**103 (20.32)**	− **0.041**	**0.02**	0.024*	− **0.037**	**0.02**	0.045*

*SE* Standard error, *CCB* Calcium channel blocker, *ACEI* Angiotensin-converting enzyme inhibitor, *ARB* Angiotensin receptor blocker, *CVD* Cardiovascular disease. *ADRs* Adverse Drug Reactions

## Discussion

### Statement of key findings

Patients with ADRs were seven times more likely to be non-adherent to their medication regimen and reported a lower quality of life compared to those without ADRs, putting them at a higher risk of suboptimal treatment outcomes. Healthcare professionals should closely monitor affected patients to mitigate the risk of CVD and other hypertension-related complications. Additional monitoring may include more routine clinical and laboratory assessments, personalized education on the importance of adherence, and proactive management of ADRs to ensure better long-term health outcomes.

### Strengths and weaknesses

To our knowledge, this is the first study to examine the association between ADRs and (i) medication adherence and (ii) HRQoL among patients with hypertension, with a thorough assessment of ADRs causality. Several sensitivity and subgroup analyses were conducted to assess the robustness of our findings, which is often overlooked in similar research. We classified non-adherence as intentional or unintentional, categorized ADRs based on severity; and explored the determinants of each HRQoL component, including mobility, self-care, usual activities, pain, and anxiety.

Nevertheless, this study also has some limitations. Due to cross-sectional nature of the study, it is difficult to establish causality between determinant and outcomes. Cohort studies may be needed to confirm temporal relationship between ADRs, medication adherence, and HRQoL. Secondly, this study used self-reported data which may introduce bias. Several studies used objective measurement for medication adherence, including estimation of medication possession ratio (MPR) and measurement of drug metabolites level [36]. Lastly, generalizability of the findings may be limited as the study was conducted in Indonesia primary care setting. Further studies conducted in other settings may be needed to confirm the findings.

### Interpretation and further research

Consistent with our result, a previous Canadian study using telephone interviews showed that patients who experienced an ADR to antihypertensive drugs were 91% more likely to decide to discontinue their medication compared to those without ADRs [37]. Our results also align with those from Berhe et al., who found that self-reported ADRs were associated with decreased adherence. However, the Berhe et al. study did not perform ADR causality and severity assessments [10]. In contrast, our study included a thorough ADR causality assessment, validated by an expert panel, along with a severity assessment and subgroup analyses based on ADR and adherence types to provide deeper insights related to the association between ADRs and medication adherence.

Although majority of the ADRs observed in this study were of mild severity, it could still have considerable impact for patients. The reactions may lead to a decreased productivity and increased emotional burden [38]. A study by Davies et al. found that patients with intolerance to multiple antihypertensive drugs had an increased risk of developing psychiatric morbidity, including anxiety, depression, and panic-related problem [39]. The lack of other studies investigating the association between ADRs and quality of life among patients with hypertension impedes direct comparison. Nevertheless, the finding in current study was in line with previous studies in different therapeutic areas, including ADRs related to antipsychotics drugs, COPD drugs, hormonal replacement therapy, antidiabetics, and oncology drugs [40–44].

In addition, our subgroup analysis found that patients with a new diagnosis of hypertension were more likely to report an anxiety-related problem as compared to those with longer duration of hypertension. Patients with a new diagnosis of chronic disease may have increased concerns about taking a life-long medication regimen [45]. Thus, it may be beneficial for these patients if their health care professionals take the time to reassure them about the strategies to address their concerns related to long-term medication use at the start of the therapy [46].

As this study showed that ADRs to antihypertensive medication were associated with poor medication adherence and reduced HRQoL, closer monitoring for patients affected by ADRs is encouraged. Additional monitoring and education can be implemented by involving patients in treatment decision-making, conducting more routine clinical and laboratory monitoring, and emphasizing the importance of medication adherence to reduce hypertension-related morbidity [47]. Pharmacists may emphasize that the benefit of antihypertensive drugs in protecting them against CVD and other morbidities due to uncontrolled hypertension outweighs the possible risks of this medication. A previous study showed that clinical decision support system application for patients and providers, which included discussing benefit and side-effects of medication had high acceptability and has the potential to improve rational use of medicines [48]. Further research may be useful to explore the impact of such interventions on managing ADRs and enhancing adherence.

## Conclusion

Patients affected by ADRs were seven times more likely to be non-adherent to their medication regimen and had a reduced HRQoL, placing them at a higher risk of suboptimal treatment outcomes. This finding calls for additional monitoring and education for patients affected by ADRs, particularly through more frequent clinical and laboratory assessments, addressing concerns and barriers related to their medication use, and emphasizing the importance of adherence to prevent hypertension-related complications.

## Supplementary Information

Below is the link to the electronic supplementary material.

Supplementary file1 (DOCX 29 kb)
