# Supplementary material for: Exploring the association of adverse drug reactions with medication adherence and quality of life among hypertensive patients: a cross-sectional study
Source: Int J Clin Pharm. 2024 Nov 28;47(2):354–64. doi: 10.1007/s11096-024-01832-9 (PMC11919996; doi:10.1007/s11096-024-01832-9)
Supplement: Supplementary file 1 — Supplementary file1 (DOCX 29 kb) [file 11096_2024_1832_MOESM1_ESM.docx]

**Table Supplementary 1. Distribution of EQ-5D Health Dimensions Problems and EQ-5D Utility Value**

| **Characteristics**  **(n=507)** | **Health Dimensions Problem (%)** | | | | | | | | | | **Mean of**  **EQ-5D score (SD)** | **P-value** |
| --- | --- | --- | --- | --- | --- | --- | --- | --- | --- | --- | --- | --- |
|  | **Mobility (n=83, 16.37** | **P-value** | **Self-Care**  **(n=17, 3.35)** | **P-value** | **Usual Activities**  **(n=74, 14.60)** | **P-value** | **Pain**  **(n=226, 44.58)** | **P-**  **value** | **Anxiety/**  **Depression**  **(n=63,**  **12.43)** | **P-value** |  |  |
| **Age** |  | 0.370 |  | 0.673 |  | 0.602 |  | 0.477 |  | 0.522 |  | 0.443 |
| ≤ 40 years | 1 (1.20) |  | 0 (0.00) |  | 3 (4.05) |  | 9 (3.98) |  | 1 (1.59) |  | 0.91 (0.20) |  |
| 41-50 years | 12 (14.46) |  | 2 (11.76) |  | 13 (17.57) |  | 35 (15.49) |  | 12 (19.05) |  | 0.87 (0.22) |  |
| 51-60 years | 24 (28.92) |  | 7 (41.18) |  | 15 (20.27) |  | 54 (23.89) |  | 20 (31.75) |  | 0.90 (0.14) |  |
| 61-70 years | 35 (42.17) |  | 5 (29.41) |  | 31 (41.89) |  | 90 (39.82) |  | 22 (34.92) |  | 0.86 (0.17) |  |
| ≥ 71 years | 11 (13.25) |  | 3 (17.65) |  | 12 (16.22) |  | 38 (16.81) |  | 8 (12.70) |  | 0.91 (0.10) |  |
| **Sex** |  | 0.808 |  | 0.188 |  | 0.811 |  | 0.051 |  | 0.030 |  | 0.064 |
| Male (%) | 22 (26.51) |  | 2 (11.76) |  | 18 (24.32) |  | 48 (21.24) |  | 9 (14.29) |  | 0.92 (0.12) |  |
| **Duration of hypertension** |  | 0.887 |  | 0.437 |  | 0.238 |  | 0.977 |  | 0.265 |  | 0.945 |
| < 1 years | 14 (16.87) |  | 4 (23.53) |  | 8 (10.81) |  | 37 (16.37) |  | 15 (23.81) |  | 0.89 (0.19) |  |
| 1-5 years | 30 (36.14) |  | 5 (29.41) |  | 33 (44.59) |  | 81 (35.84) |  | 24 (38.10) |  | 0.89 (0.15) |  |
| 6-10 years | 17 (20.48) |  | 2 (11.76) |  | 14 (18.92) |  | 55 (24.34) |  | 11 (17.46) |  | 0.90 (0.16) |  |
| > 10 years | 22 (26.51) |  | 6 (35.29) |  | 19 (25.68) |  | 53 (23.45) |  | 13 (20.63) |  | 0.89 (0.17) |  |
| **Antihypertensive drug class** |  | 0.224 |  | 0.252 |  | 0.323 |  | 0.990 |  | 0.636 |  | 0.514 |
| ***Monotherapy*** |  |  |  |  |  |  |  |  |  |  |  |  |
| CCB | 58 (69.88) |  | 16 (94.12) |  | 51 (68.92) |  | 156 (69.03) |  | 43 (68.25) |  | 0.89 (0.18) |  |
| ACEI/ARB | 2 (2.41) |  | 0 (0.00) |  | 5 (6.76) |  | 12 (5.31) |  | 2 (3.17) |  | 0.92 (0.12) |  |
| ***Combination of 2 drug classes*** |  |  |  |  |  |  |  |  |  |  |  |  |
| CCB and ACEI/ARB | 15 (18.07) |  | 0 (0.00) |  | 9 (12.16) |  | 30 (13.27) |  | 12 (19.05) |  | 0.89 (0.16) |  |
| Other 2 drugs combination | 3 (3.61) |  | 1 (5.88) |  | 3 (4.05) |  | 17 (7.52) |  | 4 (6.35) |  | 0.93 (0.08) |  |
| ***Combination of ≥ 3 drug***  ***classes*** | 5 (6.02) |  | 0 (0.00) |  | 6 (8.11) |  | 11 (4.87) |  | 2 (3.17) |  | 0.88 (0.16) |  |
| **Polypharmacy** | 18 (21.69) | 0.189 | 2 (11.76) | 0.575 | 15 (20.27) | 0.382 | 37 (16.37) | 0.832 | 13 (20.63) | 0.380 | 0.88 (0.19) | 0.500 |
| **Comorbidities** |  |  |  |  |  |  |  |  |  |  |  |  |
| Diabetes mellitus | 14 (16.87) | 0.475 | 4 (23.53) | 0.688 | 14 (18.92) | 0.851 | 45 (19.91) | 0.924 | 14 (22.22) | 0.594 | 0.89 (0.14) | 0.936 |
| Dyslipidaemia | 17 (20.48) | 0.810 | 5 (29.41) | 0.296 | 17 (22.97) | 0.418 | 52 (23.01) | 0.076 | 12 (19.05) | 0.918 | 0.87 (0.21) | 0.009 |
| Hyperuricemia | 15 (18.07) | 0.013 | 0 (0.00) | 0.152 | 9 (12.16) | 0.603 | 31 (13.72) | 0.031 | 6 (9.52) | 0.797 | 0.87 (0.13) | 0.326 |
| CVD history | 7 (8.43) | 0.986 | 0 (0.00) | 0.202 | 4 (5.41) | 0.304 | 24 (10.62) | 0.121 | 3 (4.76) | 0.258 | 0.91 (0.10) | 0.480 |
| **Education level** |  | 0.868 |  | 0.465 |  | 0.431 |  | 0.504 |  | 0.607 |  | 0.666 |
| Primary or no education | 18 (21.69) |  | 6 (35.29) |  | 21 (28.38) |  | 53 (23.45) |  | 18 (28.57) |  | 0.88 (0.19) |  |
| Secondary education | 52 (62.65) |  | 8 (47.06) |  | 44 (59.46) |  | 141 (62.39) |  | 36 (57.14) |  | 0.89 (0.15) |  |
| College/University | 13 (15.66) |  | 3 (17.65) |  | 9 (12.16) |  | 32 (14.16) |  | 9 (14.29) |  | 0.90 (0.18) |  |
| **Smoking status** |  | 0.565 |  | 0.036 |  | 0.969 |  | 0.033 |  | 0.131 |  | 0.139 |
| Current smoker | 8 (9.64) |  | 0 (0.00) |  | 8 (10.81) |  | 20 (8.85) |  | 6 (9.52) |  | 0.92 (0.15) |  |
| Former smoker | 16 (19.28) |  | 0 (0.00) |  | 12 (16.22) |  | 29 (12.83) |  | 5 (7.94) |  | 0.92 (0.11) |  |
| Never smoker | 59 (71.08) |  | 17 (100) |  | 54 (72.97) |  | 177 (78.32) |  | 52 (82.54) |  | 0.88 (0.18) |  |
| **ADRs to antihypertensive medication** | 17 (20.48) | 0.967 | 5 (29.41) | 0.343 | 16 (21.62) | 0.763 | 45 (19.91) | 0.839 | 18 (28.57) | 0.082 | 0.86 (0.24) | 0.025 |

OR: Odds Ratio. CCB: Calcium channel blocker. ACEI: Angiotensin-converting enzyme inhibitor. ARB: Angiotensin receptor blocker. CVD: Cardiovascular disease. ADRs: Adverse Drug Reactions.

**Table Supplementary 2.** **Subgroup analysis comparing determinants of each health dimension problem among**

**patients with hypertension**

| **Characteristics** | **Mobility**  **(Adjusted OR. 95% CI)** | **Self-Care**  **(Adjusted OR. 95% CI)** | **Usual Activities**  **(Adjusted OR. 95% CI)** | **Pain/Discomfort**  **(Adjusted OR. 95% CI)** | **Anxiety/**  **Depression**  **(Adjusted OR. 95% CI)** |
| --- | --- | --- | --- | --- | --- |
| **Age** |  |  |  |  |  |
| ≤ 40 years | 0.35 (0.04, 3.02) | N/A | 1.50 (0.35, 6.44) | 1.00 (0.36, 2.84) | 0.36 (0.04, 3.30) |
| 41-50 years | 1.24 (0.48, 3.18) | 0.44 (0.06, 3.31) | 1.50 (0.60, 3.79) | 1.06 (0.55, 2.06) | 1.25 (0.44, 3.52) |
| 51-60 years | 1.48 (0.65, 3.33) | 1.15 (0.24, 5.60) | 0.91 (0.39, 2.15) | 0.86 (0.48, 1.53) | 1.44 (0.57, 3.62) |
| 61-70 years | 1.70 (0.65, 3.33) | 0.60 (0.12, 3.13) | 1.43 (0.67, 3.06) | 1.35 (0.79, 2.31) | 1.26 (0.52, 3.03) |
| ≥ 71 years | 1.00 | 1.00 | 1.00 | 1.00 |  |
| **Sex, Male (%)** | 1.05 (0.51, 2.14) | 0.76 (0.13, 4.45) | 1.03 (0.49, 2.19) | 0.86 (0.50, 1.47) | 0.52 (0.21, 1.28) |
| **Duration of hypertension** |  |  |  |  |  |
| < 1 years | 1.00 | 1.00 | 1.00 | 1.00 | 1.00 |
| 1-5 years | 0.84 (0.41, 1.76) | 0.67 (0.15, 2.97) | 1.96 (0.83, 4.64) | 0.88 (0.50, 1.53) | 0.63 (0.30, 1.33) |
| 6-10 years | 0.79 (0.35, 1.76) | 0.34 (0.06, 2.12) | 1.36 (0.52, 3.54) | 0.91 (0.50, 1.66) | **0.39 (0.16, 0.93)*** |
| > 10 years | 1.00 (0.46, 2.18) | 1.07 (0.25, 4.54) | 1.96 (0.78, 4.93) | 0.84 (0.46, 1.52) | 0.52 (0.22, 1.21) |
| **Antihypertensive drug class** |  |  |  |  |  |
| ***Monotherapy*** |  |  |  |  |  |
| CCB | 0.63 (0.18, 2.17) | 3.52 (0.43, 28.71) | 0.30 (0.09, 1.02) | 0.98 (0.36, 2.66) | 1.20 (0.23, 6.39) |
| ACEI/ARB | 0.26 (0.04, 1.70) | N/A | 0.50 (0.11, 2.27) | 0.93 (0.27, 3.21) | 0.85 (0.10, 7.51) |
| ***Combination of 2 drug classes*** |  |  |  |  |  |
| CCB and ACEI/ARB | 0.87 (0.23, 3.24) | N/A | 0.29 (0.07, 1.12) | 0.91 (0.31, 2.66) | 1.74 (0.31, 9.86) |
| Other 2 drugs combination | 0.31 (0.06, 1.59) | N/A | 0.16 (0.03, 0.85) | 0.93 (0.30, 2.89) | 1.23 (0.18, 8.18) |
| ***Combination of ≥ 3 drug classes*** | 1.00 | 1.00 | 1.00 | 1.00 | 1.00 |
| **Polypharmacy** | 1.29 (0.68, 2.46) | 0.48 (0.09, 2.51) | 1.12 (0.57, 2.21) | 0.86 (0.52, 1.44) | 1.28 (0.62, 2.66) |
| **Diabetes mellitus** | 0.68 (0.35, 1.33) | 1.19 (0.33, 4.38) | 0.89 (0.46, 1.72) | 1.08 (0.68, 1.72) | 1.22 (0.61, 2.41) |
| **Dyslipidaemia** | 0.98 (0.53, 1.81) | 2.21 (0.66, 7.41) | 1.20 (0.65, 2.24) | 1.45 (0.91, 2.31) | 0.98 (0.49, 1.99) |
| **Hyperuricemia** | **2.25 (1.13, 4.48)*** | N/A | 1.10 (0.49, 2.46) | 1.73 (0.95, 3.15) | 0.85 (0.34, 2.16) |
| **CVD history** | 0.83 (0.30, 2.32) | N/A | 0.34 (0.10, 1.24) | 1.81 (0.89, 3.79) | 0.64 (0.17, 2.66) |
| **Education level** |  |  |  |  |  |
| Primary or no education | 1.00 | 1.00 | 1.00 | 1.00 | 1.00 |
| Secondary education | 1.80 (0.68, 4.79) | 0.51 (0.15, 1.78) | 0.77 (0.42, 1.42) | 1.23 (0.78, 1.94) | 0.90 (0.47, 1.72) |
| College/University | 1.35 (0.55, 3.32) | 0.86 (0.16, 4.53) | 0.57 (0.23, 1.41) | 1.00 (0.54, 1.84) | 0.82 (0.33, 2.07) |
| **Smoking status** |  |  |  |  |  |
| Current smoker | 1.00 | N/A | 1.00 | 1.00 | 1.00 |
| Former smoker | 1.80 (0.68, 4.79) | N/A | 1.34 (0.48, 3.69) | 1.10 (0.52, 2.32) | 0.63 (0.17, 2.31) |
| Never smoker | 1.35 (0.55, 3.32) | N/A | 1.16 (0.47, 2.87) | 1.76 (0.92, 3.37) | 1.08 (0.40, 2.90) |
| **ADRs to antihypertensive medication** | 1.01 (0.54, 1.88) | 2.15 (0.62, 7.45) | 1.13 (0.60, 2.16) | 0.97 (0.61, 1.54) | 1.62 (0.85, 3.08) |

OR: Odds Ratio. CCB: Calcium channel blocker. ACEI: Angiotensin-converting enzyme inhibitor. ARB: Angiotensin receptor blocker. CVD: Cardiovascular disease. ADR: Adverse Drug Reaction
